# Loss of tyrosine phosphatase SHP2 activity promotes growth of colorectal carcinoma HCT-116 cells

**DOI:** 10.1038/s41392-020-0192-0

**Published:** 2020-05-29

**Authors:** Xin Chen, Xueqi Fu, Wanke Zhao, Wan-Ting Tina Ho, Shu Xing, Zhizhuang Joe Zhao

**Affiliations:** 1 Edmond H. Fischer Signal Transduction Laboratory, College of Life Sciences, Jilin University, Changchun, China; 2 Department of Pathology, University of Oklahoma Health Sciences Center, Oklahoma City, OK USA

**Keywords:** Cell biology, Drug discovery

**Dear Editor**,

Tyrosine phosphatase SHP2/PTPN11 plays an important role in cell signaling. Activation mutation of SHP2 causes cancer, which makes the enzyme an anti-cancer drug target. Several selective and potent SHP2 inhibitors have been discovered. SHP099, the best known SHP2 inhibitor currently in clinical trials, has been shown to be effective for receptor tyrosine kinase-driven and oncogenic KRAS-driven cancers^1,2^. We employed a colorectal cancer cell line HCT-116 that carries a KRAS-G13D mutation in this study. Interestingly, SHP2 inhibitors failed to inhibit HCT-116 growth and ERK1/2 activation but in contrast had the opposite effect. We then generated SHP2 knockout cells by using the CRISPR technique and provided further evidence that loss of SHP2 activity promotes growth of HCT-116 cells in vitro and in vivo. Our study suggests that targeting SHP2 may have adverse effects on certain cancers, which may have major implications for further development of anti-cancer drugs targeting SHP2.

Colorectal cancer is the second leading cause of cancer death worldwide^3^. HCT-116 cells were derived from a patient with colorectal carcinoma and represent one of the most extensively studied cancer cell lines. The cells are known to carry the KRAS-G13D mutation according to the COSMIC database. KRAS mutations are found in 15–20% of human cancers, mostly colorectal cancer as well as in pancreatic cancer, lung cancer, and leukemia. Because of the lack of targeting therapies, KRAS mutations confer very a poor prognosis^4^. We sought to determine if SHP2 inhibitors could be potentially used to treat colorectal cancer by testing HCT-116 cells. First, by performing PCR and DNA sequencing, we verified the heterozygous KRAS-G13D mutation in HCT-116 cells at both the genomic DNA and cDNA levels (Supplementary Fig. S1). We then treated the cells with SHP2 inhibitor SHP099. Surprisingly, at a concentration as high as 10 µM, SHP099 did not suppress HCT-116 cell growth. In contrast, it moderately stimulated cell proliferation (Fig. 1a). Consistent with these results, phosphorylation of cell proliferation signaling transducer ERK1/2 and their direct upstream activation MEK1/2 also increased. To ensure that the inhibitor was effective, we also included U-2 OS osteosarcoma cells in parallel experiments and demonstrated the inhibitory effects of SHP099. Further analyses with RMC-4550, a newer and more potent derivative of SHP099, revealed consistent results (Supplementary Fig. S2). The data suggest that SHP2 inhibition has an unexpected positive role in the growth of HCT-116 cells.

**Fig. 1:**
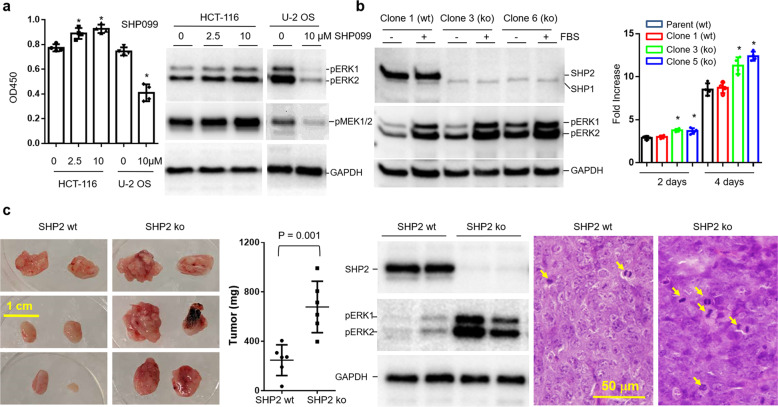
SHP2 inhibition and knockout stimulate cell signaling in HCT-116 cells and promote their growth in vitro and in vivo. **a** HCT-116 cells were cultured in the presence SHP099 for 24 h for XTT assays or 4 h for Western blotting analysis. Error bars denote SD (*n* = 4). *P < 0.001. **b** For Western blotting analysis, clonal HCT-116 cells with intact SHP2 or SHP2 knockout were serum-starved for 4 h and then treated with 10% FBS for 25 min. For growth assays, cells were cultured in medium containing 1% FBS for 2 or 4 days and then trypsinized for counting after erythrosine B staining. Error bars denote SD (*n* = 4). *P < 0.01 in reference to either of the SHP2 wild-type cells on correspondent days. **c** Immunodeficient NSG**-**SGM3 mice were subcutaneously injected with HCT-116 cells with wild-type SHP2 (clone 1) or SHP2 knockout (clone 3). Data show the size of tumors formed after 3 weeks of inoculation. Error bars denote SD (*n* = 6). Tumor tissues were subjected to Western blotting analysis and histochemical staining with hemotoxylin and eosin. Note that the two tumor tissues were embedded in the same tissue block and stained on the sample slide. The tumor resulting from SHP2 knockout cells show stronger nuclear staining with more mitotic cells (marked by arrows)

By employing the CRISPR/Cas9 genome-editing technique, we generated SHP2 knockout cells. Among the six clonal cell lines we generated, five of them displayed complete absence of SHP2 protein as revealed by Western blot analyses (Supplementary Fig. S3). DNA sequencing analyses of SHP2 transcripts revealed the deletion of a 35 bp fragment containing the translation initiation ATG site in SHP2-knockout HCT-116 cells. Importantly, knockout of SHP2 expression is accompanied by activation of ERK1/2 as indicated by their increased phosphorylation (Supplementary Fig. S3). We further treated clonal SHP2-knockout cells with SHP099 (Supplementary Fig. S4). Consistently, parental HCT-116 cells and the clonal cells with intact SHP2 responded to SHP099 with an increase in phosphorylated ERK1/2. In contrast, SHP2-knockout cells had a higher level of phosphorylated ERK1/2 without inhibitor treatment but did not show further enhancement upon treatment. This indicates that inhibition of SHP2 by SHP099 is responsible for the increased phosphorylation of ERK1/2 in HCT-116 cells with wild-type SHP2. Note that SHP099 treatment did not affect the expression level of SHP2 in wild-type HCT-116 cells.

We further investigated the response of cells to serum stimulation (Fig. 1b). Under serum-starved conditions, the level of phosphorylated ERK1/2 in SHP2-knockout cells remained higher than that in SHP2-wild-type cells. Upon serum stimulation, both types of cells displayed increased ERK1/2 phosphorylation with the knockout cells to a further extent. Note that SHP1, a phosphatase with high structural similarity to SHP2, expressed at a low level and was not affected by the Cas9-mediated SHP2 knockout. Interestingly, when cells were cultured under a sub-optimal condition with 1% fetal bovine serum, SHP2-knockout cells displayed a significantly higher growth rate (*P* < 0.01, Fig. 1b). We further investigated the growth of cells in vivo by implanting cells in immunodeficient NSG-SGM3 mice. After 3 weeks of inoculation, both wild-type and SHP2-knockout cells formed subcutaneous tumors in the mice. However, the tumors formed by the knockout cells were significant larger, suggesting an increased cell growth rate in vivo for the latter cells (Fig. 1c). As found with in vitro cultured cells, extracts of tumors from SHP2-kncokout cells displayed enhanced ERK1/2 phosphorylation. Histochemical staining of tumor sections with hematoxylin and eosin revealed higher numbers of mitotic cells among the knockout cells, further supporting the notion that SHP2-knockout HCT-116 cells possess enhanced proliferative activity.

Our present study demonstrates that loss of SHP2 activity promotes the growth of HCT-116 colorectal carcinoma cells. This finding contradicts the inhibitory effects observed in many other cancer cells^1,2^, including U-2 OS cells described in the current study. Resistance of HCT-116 to the SHP099 inhibitor is not surprising since some cells may not require SHP2 for survival and proliferation. In fact, there are reports demonstrating the resistance of certain cancer cells to SHP2 inhibitors^1^. However, our current data indicate that SHP2 inhibitors rather enhanced ERK activation in HCT-116 cells and stimulated their growth. This stimulatory effect of the inhibitor was somewhat unexpected. Evidently, this is not due to potential off-target, non-specific effects of the inhibitor because the results were further supported by SHP2 knockout cells. One may argue that HCT-116 cells may carry mutant SHP2. Interestingly, we found that HCT-116 cells predominantly express the variant v3 SHP2 isoform (Supplementary Fig. S5). In comparison with the common isoform v1, isoform v3 contains a four amino acid insertion (ALLQ) after Q408 in the catalytic domain of the SHP2 protein. The enzymatic activity of the v3 SHP2 variant has not been characterized. However, even if it displays different sensitivity toward SHP2 inhibitors, the expression of variant SHP2 isoform cannot explain the data observed with our SHP2 knockout cells. In any case, due to promising results from previous studies, targeting SHP2 represents a viable therapeutic option for receptor tyrosine kinase-driven and KRAS-driven cancers. Our study revealed a potential adverse effect of SHP2 inhibition, which should be taken into consideration in future therapeutic drug development.

It is clear that SHP2 plays both positive and negative roles in cell signaling and cell proliferation. As a highly conserved enzyme, SHP2 is present in various living organisms from unicellular protists to mammals^5^. Needless to say, certain general functions of SHP2 may be essential to life. However, as an enzyme ubiquitously expressed in various tissues in animals, its specific function in different types of cells may be diverse and largely dependent on cellular context. As a phosphatase that catalyzes protein dephosphorylation, how SHP2 exhibits a positive role in ERK activation, a process driven by cascades of protein phosphorylation events, is not clear. Likewise, since the direct substrates of SHP2 have not been identified, how SHP2 negatively regulates ERK activation needs to be investigated further. In this regard, our study provides a good cell system to address these questions.

## Supplementary information

Supplementary data

Materials and Methods

## Data Availability

The data sets used and/or analyzed during the current study are available from the corresponding author on reasonable request.
