# Supplementary material for: Loss of tyrosine phosphatase SHP2 activity promotes growth of colorectal carcinoma HCT-116 cells
Source: Signal Transduct Target Ther. 2020 May 29;5:83. doi: 10.1038/s41392-020-0192-0 (PMC7256044; doi:10.1038/s41392-020-0192-0)
Supplement: Supplementary file 1 — Supplementary data [file 41392_2020_192_MOESM1_ESM.docx]

**Supplementary data**

**Fig. S1. Verification of the heterozygous KRAS G13D mutation in HCT-116 cells at the genomic and cDNA level by Sanger DNA sequencing.** DNA fragments containing the entire exon 1 and the full-length coding sequence of KRAS were PCR-amplified from genomic and single-strand cDNA obtained from HCT-116 cells, respectively. Sanger DNA sequencing with 5’ PCR primers revealed heterozygous GGC-to-GAC mutation that results in amino acid G13 to D substitution as indicated.

**Fig. S2. SHP2 inhibitor RMC-4550 stimulates cell signaling in HCT-116 cells and their growth.** HCT-116 and U-2 OS cells were cultured in the presence or absence of RMC-4550. For cell proliferation assay (left panel), cells were incubated with the indicated concentrations of inhibitors for 24 hr. Relative cell proliferation was assessed by performing XTT assays. Error bars denote standard deviation (n = 3). *P < 0.001 in reference to correspondent controls. For cell signaling assay (right panel), cells were extracted in SDS gel sample buffer after 4 hr of exposure to the inhibitor and then subjected to Western blotting with anti-pERK1/2, pMEK1/2, and GAPDH as indicated.

**Fig. S3. Knockout of SHP2 in HCT-116 cells caused increased ERK activation.**  **a.** HCT-116 cells were transfected with CRISPR constructs as described in Materials and Methods. Clonal cells were selected and further expanded. Data reveal knockout of SHP2 expression in clones 2-6 but not in clone 1 that exhibited a similar level of SHP2 expression as detected in the parent cells (Wt). Activity of cell growth signal transducer ERK1/2 was detected by using phospho-specific anti-ERK1/2 antibody, while equal protein loadings were illustrated by staining of Western blotting membrane with the Ponseau S dye. **b.** DNA sequencing analyses of SHP2 transcripts (from reverse direction) in wild type and SHP2-knockout HCT-116 cells reveals deletion of a 35 bp fragment (underlined in the wild type sequence) in the position marked by a triangle.

**Fig. S4. SHP2 inhibitor SHP099 fails to enhance ERK1/2 activation in SHP2-knockout HCT-116 cells.** Parental and clonal HCT-116 cells were treated with 10 µM SHP099 for 4 hr. Cells were extracted in SDS gel sample buffer for Western blotting with anti-pERK1/2 and GAPDH. Note that the level of pERK1/2 is higher in SHP2-knockot cells than in cells with intact SHP2, but treatment with SHP099 did not enhance the pERK1/2 level in SHP2-knockout cells.

**Fig. S5. HCT-116 cells predominantly express a variant form of SHP2 with an insertion in the catalytic domain.** A DNA fragment containing the entire coding region of SHP2 was PCR-amplified from single-strand cDNA obtained from HCT-116 cells. Sanger DNA sequencing revealed that the PCR product corresponds to the variant isoform v3 (NM_001330437.2) of SHP2. In comparison with the common isoform v1, isoform v3 contains a 12 bp insertion (GCTCTACTCCAG, underlined) resulting in a 4 amino acid insertion (ALLQ) after Q408 in the catalytic domain of the SHP2 protein.
