# Supplementary material for: Loss of tyrosine phosphatase SHP2 activity promotes growth of colorectal carcinoma HCT-116 cells
Source: Signal Transduct Target Ther. 2020 May 29;5:83. doi: 10.1038/s41392-020-0192-0 (PMC7256044; doi:10.1038/s41392-020-0192-0)
Supplement: Supplementary file 2 — Materials and Methods [file 41392_2020_192_MOESM2_ESM.docx]

**Materials and Methods**

**Reagents**

Allosteric SHP2 inhibitors SHP099 and RMC-4550 were purchased from ChemieTek (IN, USA) and dissolved in dimethyl sulfoxide (DMSO) for cell-based assays. Monoclonal antibodies against phospho-ERK1/2 (pT202/pY204), phospho-MEK1/2 (Ser217/221), and GAPDH were obtained from Cell Signaling Technology (MA, USA). Monoclonal anti-SHP1 and anti-SHP2 antibodies were obtained from BD Biosciences.

**PCR and DNA sequencing analysis.**

Genomic DNA was isolated from HCT-116 cells by phenol/chloroform extraction following proteinase K digestion. A DNA fragment covering the first exon of the KRAS gene was PCR amplified with primers CATTTTTCTTAAGCGTCGATGGAGG and GCACAGAGAGTGAACATCATGGACC. Total RNA was isolated from the cells by using the Trizol reagent (Invitrogen) and reverse transcribed with the QuantiTect reverse transcription kit from Qiagen. DNA fragments covering the entire coding regions of KRAS, p53, and SHP2 were amplified by PCR using primer pairs TCCCAGGTGCGGGAGAGAGG/AAAGCTAACAGTCTGCATGGAGCAG, GCCAGACTGCCTTCCGGGTCACT/AGAGATGGGGGTGGGAGGCTGTC, and AGCCCGATGTGACCGAGCCC/ GGGAGAGGGTGAAAGTCCACATCTATTTC, respectively. PCR products were gel-purified and then subjected to Sanger sequencing. Sequencing data was analyzed by using the Chromas program.

**Cell culture and generation of SHP2 knockout HCT-116 cells.**

HCT-116 and U-2 OS cells were obtained from ATCC (Manassas, VA, USA) and cultured in DMEM containing 10% fetal bovine serum (FBS) in a humidified atmosphere at 37°C with 5% CO_2_. Knockout of SHP2 in HCT-116 cells was achieved by using the CRISPR-Cas9 system following an established protocol. In brief, two sets of guide DNA primers targeting the first exon of SHP2 with sequences of AGCAAGGAGCGGGTCCGTCG and GGAGGAACATGACATCGCGG were synthesized and cloned into the pSpCas9(BB)-2A-Puro (PX459) V2.0 vector. The resulting plasmid DNAs were then verified by DNA sequencing and used to co-transfect HCT-116 cells using the Fugene 6 cell transfection reagent. Transfected cells were then treated with 2 µg/ml puromycin and further subjected to clonal selection. Positive clones were verified by performing Western blotting analysis with anti-SHP2 antibody and expanded in DMEM with 10% FBS for further analyses.

**Cell growth and signaling assays**

Cells were incubated with different concentrations of inhibitors for up to 4 days. Stock solutions of inhibitors were made in DMSO, and the final concentration of DMSO in the cell incubation system was controlled at 0.2%. For cell growth analysis, viable cells were enumerated after Erythrosin B staining with a cytometer. Cell proliferation was also assessed by performing XTT assays as previously described. In brief, cells were incubated with 0.08 mg/ml XTT (2,3-Bis-(2-Methoxy-4-Nitro-5-Sulfophenyl)-2H-Tetrazolium-5-Carboxanilide) 8 µM Phenazine Methyl Sulfate (PMS) were added to the cells, and absorbance at 450 nm was measured after 3 hr incubation at 37°C. Cell signaling was analyzed by performing Western blot analysis. Inhibitor-treated or non-treated cells were washed with cold phosphate-buffer saline and extracted in 1X SDS gel sample buffer. Cell extracts containing equal amounts of total proteins were separated by 10% SDS-PAGE and transferred to polyvinylidene difluoride membranes. The membranes were probed with a primary antibody and then with a horseradish peroxidase-conjugated secondary antibody. Detection and quantification of protein bands were carried out by using the FluorChem SP enhanced chemiluminescence imaging system from Alpha Innotech. One set of data representing at least three independent experiments are shown.

**Inoculation of tumor cells in immunodeficient mice.**

Immunodeficient NSG-SGM3 mice were purchased from Jackson laboratory (ME, USA) and kept under specific pathogen-free conditions with sterilized food and tap water given *ad libitum*. Wild type and SHP2-knockout HCT-116 cells were cultured to confluency and collected by trypsinization. After washing with cultured medium, 1×10^6^ cells in 0.1 ml complete medium were subcutaneously implanted in the flank of mice (10 weeks old, male). Mice were monitored on a daily basis and were euthanized after three weeks when the largest tumor reached about one centimeter in diameter. The animals were euthanized in a CO2 chamber connected to CO2 tanks. This was followed by open chest surgery to collect tissues for further analysis. Tumor tissues were subjected to fixation with formalin for histochemical staining and extraction in SDS gel sample buffer for Western blot analysis as described above. Sections (5 μm) were cut and stained with hematoxylin and eosin. The animal experiments were carried out under approved protocols.

**Statistical analyses**

Statistical analyses were carried out by using the Graphpad Prism software. A two-tailed t test was performed to compare the difference between two groups. A P value of less than 0.05 was considered statistically significant.
